# Health worker preferences for community-based health insurance payment mechanisms: a discrete choice experiment

**DOI:** 10.1186/1472-6963-12-159

**Published:** 2012-06-14

**Authors:** Paul Jacob Robyn, Till Bärnighausen, Aurélia Souares, Germain Savadogo, Brice Bicaba, Ali Sié, Rainer Sauerborn

**Affiliations:** 1 University of Heidelberg, Institute of Public Health, Heidelberg, Germany; 2 Department of Global Health and Population, Harvard School of Public Health, Cambridge, USA; 3 Africa Centre for Health and Population Studies, University of KwaZulu-Natal; 4 Nouna Health Research Centre, Ministry of Health, Ouagadougou, Burkina Faso; 5 Nouna Health District, Ministry of Health, Ouagadougou, Burkina Faso

**Keywords:** Health insurance, Health workers, Third-party payers, Choice behaviour, Burkina Faso

## Abstract

**Background:**

In 2004, a community-based health insurance scheme (CBI) was introduced in Nouna health district, Burkina Faso. Since its inception, coverage has remained low and dropout rates high. One important reason for low coverage and high dropout is that health workers do not support the CBI scheme because they are dissatisfied with the provider payment mechanism of the CBI.

**Methods:**

A discrete choice experiment (DCE) was used to examine CBI provider payment attributes that influence health workers’ stated preferences for payment mechanisms. The DCE was conducted among 176 health workers employed at one of the 34 primary care facilities or the district hospital in Nouna health district. Conditional logit models with main effects and interactions terms were used for analysis.

**Results:**

Reimbursement of service fees (adjusted odds ratio (aOR) 1.49, p < 0.001) and CBI contributions for medical supplies and equipment (aOR 1.47, p < 0.001) had the strongest effect on whether the health workers chose a given provider payment mechanism. The odds of selecting a payment mechanism decreased significantly if the mechanism included (i) results-based financing (RBF) payments made through the local health management team (instead of directly to the health workers (aOR 0.86, p < 0.001)) or (ii) RBF payments based on CBI coverage achieved in the health worker’s facility relative to the coverage achieved at other facilities (instead of payments based on the numbers of individuals or households enrolled at the health worker’s facility (aOR 0.86, p < 0.001)).

**Conclusions:**

Provider payment mechanisms can crucially determine CBI performance. Based on the results from this DCE, revised CBI payment mechanisms were introduced in Nouna health district in January 2011, taking into consideration health worker preferences on how they are paid.

## Background

In early 2004, a community-based health insurance (CBI) scheme, *Assurance Maladie à Base Communautiare (AMBC)*, was introduced in Nouna health district, Burkina Faso. CBI is a common term used for voluntary, not-for-profit health insurance schemes, organized at the level of the community [1,2]. Under CBI schemes, members of a community, often defined by geographical proximity or through employment-based relationships, pool resources in order to provide support for covering health expenditures [3]. CBI has been seen as an attractive solution to the challenge of generating financial resources for healthcare in developing countries, because CBI is designed to assist the many people in those countries who work in rural and informal sectors. Such people rarely have access to other types of health insurance, since these usually require employment in the formal sector [2,4-8]. The development of CBI programs in sub-Saharan Africa has garnered substantial interest by both researchers and policymakers alike, as an instrument to reduce financial barriers to care where other types of health insurance schemes cannot be implemented [7,9-17]. Two important challenges in establishing and sustaining CBI schemes are low rates of community member enrollment and high dropout rates, leading to low CBI coverage. Low CBI coverage, in turn, results in low levels of revenue and limited risk-pooling, which can leave CBI schemes financially and organizationally vulnerable to unexpected changes in enrollees’ incomes or disease incidence.

### Study setting

This study took place in Nouna health district in northwest Burkina Faso, is a predominantly rural area where the majority of the population depends on subsistence agriculture as their primary livelihood. The city of Nouna is approximately 300 km from Ouagadougou (the capital of Burkina Faso) and approximately 100 km from the border with Mali. The city is both the headquarters of Nouna health district as well as the administrative center of the province of Kossi.

The details of the implementation of the Nouna CBI scheme and its benefit package are described elsewhere [18-20]. The participation of health workers in the scheme depends on whether they are employed in the CBI implementation zone or not. Facilities that operate within the CBI implementation zone sign two-year contracts with the insurance scheme, in which the mechanism and schedule for provider payments for coverage of enrollees’ expenses are defined. At the time of the study (April/May 2010), all 13 primary-care facilities and the one secondary-care facility (the district hospital) within the zone, in which the CBI has been implemented, contracted with the scheme. In addition to these 13 facilities, 21 primary-care facilities operated within Nouna health district but were outside of the CBI scheme’s implementation zone.

### Description of the existing provider payment mechanisms of the Nouna CBI

**Figure 1:**
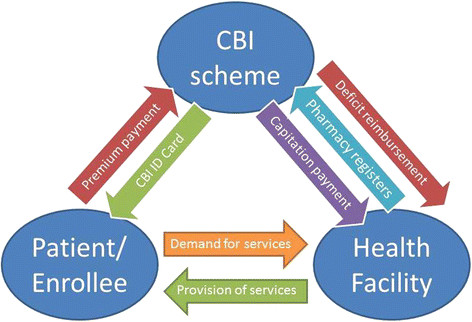
Description of the Nouna CBI financing and payment model, 2004–2010.

**Figure 2:**
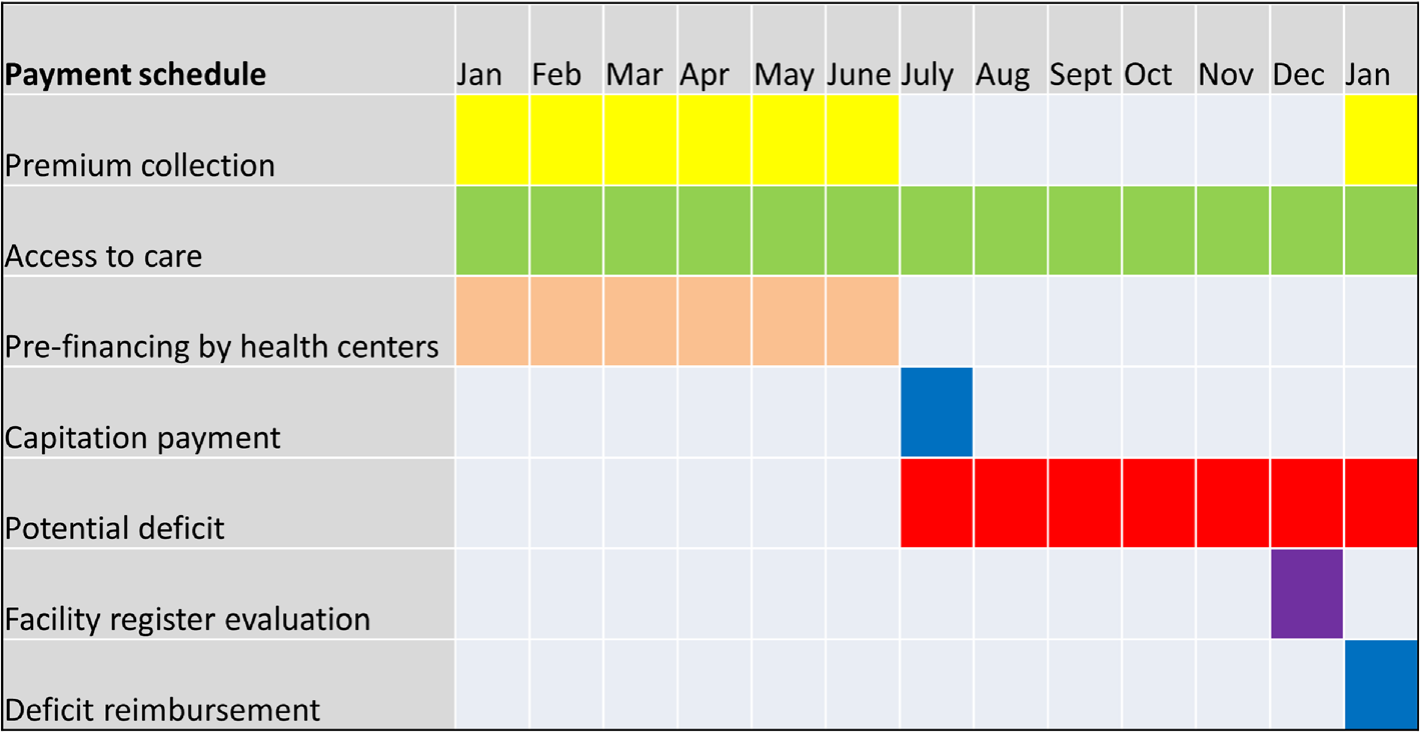
Description of the Nouna CBI's health facility payment schedule, 2004–2010.

At the time of the study, the CBI scheme in Nouna used a third-party payment mechanism to finance care provided to the scheme’s enrollees (see Figures 1 and 2). Within this payment mechanism, primary- and secondary-care facilities, which had contracted with the scheme, were paid by the CBI on an annual capitation basis, i.e., the facilities received a flat payment per individual enrolled in the CBI [4,21]. These payments were only intended to cover the cost of drugs prescribed to enrollees by health facility personnel. Premiums paid by households were collected during the annual enrollment campaign (January-June each year). If individuals were enrolling for the first time, they were obligated to adhere to a three-month waiting period before receiving their CBI ID card, which granted them access to services and drugs included in the scheme’s benefit package [18,22]. If individuals enrolled during the previous year, their ID card was automatically updated and no waiting period was enforced.

Once the campaign closed at the end of June, the CBI Management Unit calculated the level of capitation payments that would be made to primary care facilities and the district hospital. Health facility payments were based on the number of individuals who enrolled in the catchment areas of each primary care facility. Once the total premium revenue for each facility was calculated, 10% of funds were set aside for operational costs of the scheme. Of the remaining 90%, 75% was allocated to the associated primary care facility and 25% to the district hospital.

Pharmacy registers were provided to each primary care facility and the district hospital, and were used to track drugs prescribed to CBI enrollees over the length of the calendar year. The pharmacy manager of each facility was given the task to update registers as enrolled patients were provided drugs. At the end of each calendar year, the total costs incurred through enrollee prescriptions were calculated. If the annual total exceeded the sum allocated through the initial capitation payments, the financial deficit was reimbursed by an external fourth party (since 2005, a philanthropic German foundation) during the first quarter of the following year. Service fees, such as consultation and medical service fees, were not included in this reimbursement, nor were they paid by enrolled patients. Further details of the CBI payment mechanism at the time of the study are shown in Table 1.

**Table 1:** CBI payment mechanisms, 2004-2010

**Payment attribute**	**Explanation**
Capitation payment level	The enrollment premium was 500 FCFA ($1 USD) for children under 15 years of age and 1500 FCFA ($3 USD) for adults 15 years of age and older. 10% of the total of capitation payments was reserved for the CBI management, and the remaining 90% was split between primary- (3 quarters) and secondary-care (1 quarter) facilities. The capitation payment was meant to cover all drug costs for enrollees during the calendar year.
Capitation payment schedule	The capitation was paid once a year, normally in July or August, after the annual enrollment campaign closed (end of June each year).
Allocation of medical supplies and equipment	Neither medical supplies (cotton, alcohol, Bétadine, Sparadrap, etc.) nor medical equipment (tension meter, stethoscope, thermometer, scale, height gauge) were paid for by the CBI scheme.
Reimbursement of consultation and service fees	None. The capitation paid to facilities was meant to cover only the cost of drugs prescribed to enrollees. Fees for consultations and services consumed by enrollees were not covered by the annual capitation, nor included in the calculations to determine the annual deficit reimbursement (see below), and were not paid by CBI enrollees.
Capitation deficit reimbursement	If the total cost of drugs prescribed to enrollees exceeded the capitation payment, the resulting deficit for each calendar year was reimbursed (by an external fourth party) during the first quarter of the following calendar year.
Results-based financing (RBF) provider payment mechanism	At the time of the study, there existed no results-based financing provider payment mechanism (financial or non-financial) linked to CBI coverage.

FCFA: Franc Communauté Financière Africaine, the local currency used in Burkina Faso. 500 FCFA = $1 USD.

CBI: Community-based health insurance.

The financing and payment mechanisms used by the Nouna CBI scheme were somewhat of a radical departure from those found within the traditional approach to financing public sector health facilities in Burkina Faso. In the traditional financing mechanism, health facilities acquired funds through two general sources: (i) budget allocations from the Ministry of Health, and (ii) revenue generated from service fees and drug sales. The second source of revenue was used for minor facility investments, the restocking of drugs and supplies, and health worker bonuses. A significant proportion of service fee revenue (20-22%) was reserved for the bonuses (known as *ristournes*), which were paid on a quarterly basis. Within these traditional financing and payment mechanisms, individual health workers had two primary sources of income: (i) a monthly salary, and (ii) the abovementioned quarterly bonus, which had the potential to be a considerable source of income for health workers stationed in facilities with high utilization rates. Under the Nouna CBI's provider payment mechanism, enrolled patients no longer paid service fees, and capitation payments were only made to cover the cost of drugs prescribed to enrollees. For health facilities where a significant proportion of patients were CBI enrollees, the fact that service fees were not paid by enrollees (nor reimbursed by the CBI scheme), constituted a significant loss in revenue for the health facility and the workers employed there [23,24].

Previous unpublished studies on the CBI scheme in Nouna have found wide-spread health worker dissatisfaction with the CBI payment mechanism, noting their particular dissatisfaction with various payment attributes [23,25]:

· The low overall level of capitation (which in the past has led to budget deficits that needed to be covered by an external donor);

· The payment schedule (once per year in July, leading to facilities pre-financing enrollee medical costs during the first six months of the budget cycle);

· The fact that capitation is the only payment mechanism used by the CBI scheme (when additional payment mechanisms could possibly improve health worker motivation and the financial situation of facilities).

Low health worker satisfaction, inappropriate incentive structures, and fear of facility bankruptcy have led to health worker resistance to provide friendly, comprehensive, and high-quality care to CBI enrollees [23,25]. In turn, the perception in the community that CBI enrollees receive worse-quality care than other patients (e.g., less friendly reception by health workers and lower quantities of drugs) is likely to have been a major cause of the low CBI coverage observed [23,25]. Studies in other settings have also shown that provider payment mechanisms are indeed an important factor affecting CBI coverage, because they crucially determine health-worker satisfaction and support for a CBI [13,26].

Since the inception of the CBI scheme in Nouna, coverage has remained low, despite an upward trend over time [27,28]. During the first year of operation (2004) coverage was 5%; by 2010, coverage had merely increased to 9%. Enrollee drop-out rates have also been high throughout the existence of the CBI, despite a decline over time (the annual drop-out was 32% in 2004 and 16% in 2010) [29]. A study in 2006 found that the most common reasons for dropping out of coverage included several that were linked to health worker attitudes and behaviors, such as “I didn’t like medical staff behavior” (19%), “Not satisfied with services received” (7%), and “I was not given good drugs” (6%) [30], suggesting that improved health worker support of the CBI could reduce enrollee drop-out. Again, based on previous studies, it seems likely that health worker support for the CBI could be substantially improved through changes in payment mechanisms, which in turn would reduce drop-out rates [23,25].

Provider payment may not only improve CBI coverage indirectly through influence on health worker satisfaction and motivation, but also directly. Results-based financing (RBF) linked to CBI coverage has the potential to motivate health workers to increase the number of people enrolled in CBI. In essence, RBF involves the “transfer of money or material goods conditional on taking a measurable action or achieving a predetermined performance target” [31]. Within the context of the CBI scheme in Nouna health district, there is substantial capacity for health workers to do more to promote CBI. According to patient exit interviews in 2010, in only 8% of healthcare visits of non-enrollees did health workers mention the possibility to enroll in CBI, and in only 3% of healthcare visits of enrollees did they remind patients to re-enroll in the CBI [32]. By introducing an RBF mechanism that is explicitly linked to changes in CBI coverage, health workers could be financially motivated to promote enrollment in the CBI.

In this study, we investigate health worker preferences for CBI payment mechanisms. We use a discrete choice experiment (DCE) to examine CBI provider payment attributes that influence health workers’ stated preferences for an insurance payment mechanism that incorporates not only payment level, timing and reimbursement options, but also a results-based financing mechanism linked to CBI coverage. We test the premise that provider payment mechanisms currently applied by the CBI scheme are poorly aligned with health worker preferences for how they are paid. We hypothesize that the misalignment of incentives has led to poor levels of provider satisfaction, inducing a resistance to support the CBI scheme’s efforts to improve enrollment levels. By revising the payment mechanism and aligning provider incentives with CBI objectives, health worker satisfaction with the scheme may improve, leading to increased support for the scheme and in turn to an increase in the number of health workers promoting enrollment. This study specifically explores payment attributes that are amenable to change, to assist policy makers in re-designing the provider payment mechanisms used by a CBI, with the aim to increase health workers’ motivation to support and promote CBI.

## Methods

### Study sample

As the CBI scheme aims to extend its zone of operation from 13 to all 34 primary-care facilities in Nouna health district by 2013, our study sample included all 185 health workers employed at one of the primary- and secondary-care facilities in the district. Of these health workers, 105 (57%) were employed at facilities within the CBI implementation zone at the time of data collection.

### Theories underlying DCE

Several theories underlie DCEs and the analysis of DCE results. DCEs are consistent with Lancaster’s theory of consumer demand [33], in which consumers have preferences for and derive utility from the underlying attributes of goods, rather than actual goods per se. DCEs are also consistent with choice-based consumer theory in that they explicitly assume that choices observed reveal the preferences of individuals [34]. Choices made in DCEs are analyzed using random utility theory [35], which assumes that utility *(U)* for individual *i* conditional on choice *j* can be decomposed into an explainable systematic component *V*_*ij*_ and a random component [36]:

(1) $$U_{\mathrm{ij}}=V_{\mathrm{ij}}+\epsilon_{\mathrm{ij}},j=1,\ldots ,J$$

The random component captures unobservable attributes of the goods, unobserved preference variation, systematic error and random measurement error. The systematic component is a function of observed attributes of the good or service and observed characteristics of individuals who make choices, which can be modeled as followed:

(2) $$P(Y_{1}=1)=P(U_{i1}>U_{\mathrm{ij}})$$

(3) $$=P(V_{i1}+\epsilon_{i1}>V_{\mathrm{ij}}+\epsilon_{\mathrm{ij}})$$

(4) $$=P(V_{i1}-V_{\mathrm{ij}}>\epsilon_{\mathrm{ij}}-\varepsilon_{\mathrm{i1}})\forall j\neq 1$$

where *Y*_*i*_ is a random variable denoting the choice outcome. Estimable choice models are then derived by assuming a joint probability distribution for the random component [36].

### DCE design

In order to determine how to divide the CBI payment mechanisms into coherent attributes that could be easily understood by respondents, we conducted 6 focus-group discussions and 16 in-depth interviews with health workers practicing within the CBI zone, as well as 3 in-depth interviews with members of the CBI Management Unit. Based on analyses of these qualitative findings [25], we produced a list of 10 candidate payment attributes, which was presented to the CBI Management Unit and District Health Management Team (DHMT) for discussion. In order to make sure that there was no overlap in attributes, and to ensure that the proposed attributes comprehensively described the CBI payment mechanisms, the list was also presented to local stakeholders during a half-day workshop. During this workshop, the final list was narrowed to 6 attributes and then validated by workshop participants as being a representative description of the CBI payment mechanisms. Four attributes on the final list were related to the provider payment mechanisms currently in place and two attributes were related to a proposed RBF mechanism that would pay health facilities an additional bonus payment based on CBI coverage results for each health facility. The final DCE attributes included: (1) the level of capitation paid, (2) the capitation payment schedule, (3) medical supplies and equipment paid for by the CBI scheme, (4) reimbursement of service fees, (5) the indicator used to determine the size of the RBF payment, and (6) the recipient of the RBF payment. Follow-up meetings were held with the CBI Management Team and DHMT to choose and validate the levels for the 6 final DCE attributes. For each attribute, either two or three levels were chosen, with the baseline level for the first four attributes being the payment mechanism at the time of the study (Table 2).

**Table 2:** DCE insurance payment attributes and levels

**Number**	**Payment element**	**Level**	**Payment modality**
**1**	Level of capitation payment per individual	A	500 FCFA per child (under 15 years of age) and 1500 FCFA per adult (*current level*)
		B	500 FCFA per child (under 15 years of age) and 1500 FCFA per adult. Children will continue to pay 500 FCFA, while a 1000 FCFA subsidy (financed by an external fourth party) will be added for payment to facilities for each child enrolled
**2**	Capitation payment schedule	A	Payment one time per year (*current schedule*)
		B	Payment twice per year (each April and July)
		C	Payment four times per year (each quarter)
**3**	Annual allocation of medical supplies/equipment by CBI scheme	A	None (*current allocation*)
		B	Basic medical supplies (cotton, alcohol, Bétadine, Sparadrap)
		C	Basic medical supplies (cotton, alcohol, Bétadine, Sparadrap) and medical equipment (tension meter, stethoscope, thermometer, scale, height gauge)
**4**	Reimbursement of service fees (consultation + medical acts), financed by an external fourth party	A	None (*current reimbursement*)
		B	Reimbursement at 50% the price of service fees paid by non-enrollees
		C	Reimbursement at 100% the price of service fees paid by non-enrollees
**5**	Results-based financing (RBF) – indicator to determine size of payment	A	By individual enrolled (500 FCFA for new enrollees and 250 FCFA for re-enrollees)
		B	By household enrolled (2000 FCFA for newly enrolled households and 1000 FCFA for households who renew their membership)
		C	A monetary award for the three best health facilities, based on the increase in CBI coverage between the previous and the current year
**6**	Results-based financing (RBF) – recipient.	A	Individual health agents (distribution of RBF among different team members will be pre-determined and applied district-wide)
		B	Global payment for health worker team (method of RBF distribution among different team members will be decided by the facility team members)
		C	Local health facility management committee will decide on method of RBF distribution among health workers and facility needs

FCFA: Franc Communauté Financière Africaine CFA, the local currency used in Burkina Faso. 500 FCFA = $1 USD.

Payment levels for the capitation attribute (#1) were determined based on an ongoing local policy debate on whether a fourth external party (the same German philanthropic organization mentioned above) would subsidize child premiums with an additional 1000 Franc Communauté Financière Africaine (FCFA), which equals about $2 USD, in order to be equal to the 1500 FCFA premium paid by adults. Previous simulations had been run with various subsidy levels and the additional 1000 FCFA was estimated to significantly reduce the recurring annual deficit. Given that health centers were obligated to use service fee and drug sale revenues from uninsured patients to cover short-term deficits created by enrollee drug consumption levels, annual in-kind drug or medical supply contributions provided directly by the CBI scheme was included as a provider payment attribute (#3). It was also decided that the mechanism for RBF payments would not replace any existing financing mechanisms, but would act as a top-up for capitation payments and would be directly linked to facility-level CBI enrollment outcomes. The RBF payment levels proposed in attribute #5 were based on the CBI scheme’s budget limitations but also took into consideration what would be considered sufficient to motivate health workers given current enrollment rates.

The six attributes produced a full factorial of 486 possible alternatives. A blocked design was applied to create 10 questionnaire versions, where versions were generated by randomly assigning (without replacement) choice sets from the overall design. Each choice set included two non-labeled payment mechanism alternatives without an opt-out option (being able to choose the current payment mechanism). While health applications generally have used smaller numbers of choice sets; in other fields of research 32 choice sets or more per respondent have commonly been employed [36]. Given the relatively small number of health workers employed in Nouna health district at the time of the survey (less than 200), and the fact that respondents were not obligated to complete the questionnaire in one sitting (respondents had 10 days to complete the questionnaire before submitting to the District Health Office), we decided to use a comparatively large number of choice sets. Choice sets were selected using an experimental design developed in STATA 11 that ensured both balance (i.e., levels of each attribute appear equally often) and orthogonality (i.e., all attributes are statistically independent of one another) of the attributes. This selection approach also minimized overlap among attribute levels (i.e., attribute levels do not repeat themselves within choice sets) and maximized utility balance (i.e., different alternatives within choice sets have similar probabilities of being chosen). These properties are desirable design criteria, allowing for maximum estimation efficiency [36,37]. Each block version was randomly assigned to 20 respondents, as empirical evidence shows that rarely more than 20 respondents per survey version are needed to estimate reliable models using discrete choice data [36]. Respondents were then asked to select their preferred payment mechanism from each of 21 choice sets (20 random and 1 fixed). The fixed choice set offered two alternative payment mechanisms, with one intended to be strictly dominant over the other.

The survey questionnaire included three sections. In the first section, information was collected on respondents’ demographic and professional characteristics, including age, sex, ethnicity, current professional title and qualifications, years worked at current facility and within Nouna health district, and current employment at a healthcare facility within the CBI implementation zone *versus* outside the zone. The second section presented the 20 randomized choice sets and 1 fixed task. In the third section, respondents were asked to simply choose their preferred payment option for each attribute included in the DCE.

The full questionnaire was pre-tested with 10 health workers and minor revisions were made, including changes to the terminology used to describe several attribute levels and the addition of a more detailed description of the objectives of the study and the history of the Nouna CBI scheme. During the data collection process, the research team visited all 34 primary-care facilities and the 10 specialty services at the district hospital. All respondents took part in a detailed presentation on the CBI scheme and its payment mechanisms, as well as how to complete the questionnaire. First, the contextual background for the study was presented to participants, noting that the CBI scheme management aimed to reform its provider payment system in 2011, and would like to better understand health worker preferences before making any changes. The current CBI payment mechanism, particularly the breakdown of how capitation payments were derived from premiums, was then described in detail, followed by a presentation on the payment attributes and corresponding levels found in the questionnaire. For many participants, it was their first exposure to RBF, so attention was paid to explaining carefully and repeatedly the “linking payments to results” approach of RBF. One practice choice set was presented to each participant, who completed it in the presence of an interviewer, who was available to assist if questions arose. Questionnaires were then independently completed and submitted to the District Health Office within 10 days from the research team’s visit.

### Statistical analysis

After data entry, plausibility checks and data cleaning, we estimated sample summary statistics in STATA 11. As the response data for the selection of alternative payment mechanisms was a dichotomous outcome (1 = being chosen; 0 = not being chosen), dummy coding was used to transform the L attribute levels into L-1 dummy variables in which each dummy is set equal to 1 when the qualitative level is present and set equal to 0 if it is not. We estimated main-effects conditional logit models with payment-mechanism attributes as the sole explanatory variables using STATA’s *clogit* command. The model allowed us to estimate how the choice among alternative mechanisms is affected by characteristics of the mechanisms that vary across choice sets. The conditional logit model is an appropriate model when data includes both chosen alternatives and alternative-specific regressors [38], as is the case with DCE data. In the conditional logit model, the predicted probability of observing outcome *m* is:

(5) $$P(y_{i}=m|z_{i})=\frac{\exp(z_{\mathrm{im}}\gamma )}{\Sigma_{j=1}^{j}\exp(z_{\mathrm{ij}}\gamma )}for\;m=1\;to\;J$$

where *z*_*im*_ contains values of the independent variables for the alternative *m* for case *i*.

For the alternative-specific regressors, the odds ratio is the multiplicative effect of being offered the selected attribute level relative to the baseline level on the odds of choosing any given payment mechanism alternative [38].

To understand how respondents’ demographic and professional characteristics influenced their payment preferences, we also estimated models that included interaction terms for several variables with payment attribute variables. To test how gender influenced payment preferences, we included sex (1 = male; 0 = female) in Model 2. Given that head nurses had substantially more involvement in the financial aspects of health facility management, we included an interaction term for the respondent’s title at the health facility in Model 3 (1 = head nurse; 0 = other). To examine if preferences significantly differed between health workers at primary care facilities and those working at the district hospital, we included level of care where the respondent was employed in Model 4 (1 = primary-care facility; 0 = secondary-care facility). Finally, in order to understand how prior experience with CBI influenced respondents’ preferences, we included facility location in Model 5 (1 = CBI intervention zone; 0 = outside CBI intervention zone). Inclusion of interaction terms in Models 2–5 also allowed for sensitivity analysis, allowing us to investigate the robustness of our main findings in comparison to the main-effects model (Model 1).

We repeated the analysis using a conditional logit model without the respondents who chose the payment mechanism that was intended to be inferior in the fixed task choice set, as it is plausible (but not cogent) that these respondents gave invalid answers. Finally, to ensure that the most appropriate model was used, we repeated the statistical analysis for the main effects model using a random-effects logit model (Model 6), a fixed-effects logit model (Model 7), and a random-effects probit model (Model 8).

### Ethics

The University of Heidelberg received approval for the research from their human subjects committee in Germany (130/2002) which was extended in 2005 and 2008, as well as the Nouna Health Research Center ethical committee (2005-005/CLE/CRSN). All respondents were informed of the research objectives and were asked to take part in the study. Those who agreed were asked to sign a consent form.

## Results

Out of 185 healthcare workers in Nouna district, 176 (95%) participated in the survey. Three respondents refused to participate, and six were absent during the data collection period. Demographic and professional characteristics of the sample are presented in Table 3.

**Table 3:** Socio-professional characteristics of respondents

**Characteristic**	**Value**	
	**No.**	**%**
Respondents	176	100
**Sex**		
Male	103	59
Female	73	41
**Age**		
< 30	44	25
30-34	77	44
35-40	36	20
40-44	9	5
45-50	6	3
> 50	4	2
**Ethnicity**		
Mossi	69	39
Bwaba	27	15
Samo	16	9
Dafing	14	8
Gurunsi	11	6
Other	39	22
**Current work location**		
Based at first-line facility (CSPS)	107	62
Based at second-line facility (CMA)	69	39
Facility in current CBI zone	101	57
Facility outside current CBI zone	75	43
**Current professional title**		
Doctor	4	2
Professional nurse with specialization (AS)	10	6
Facility head nurse (ICP)	32	18
Professional nurse with diploma (IDE)	19	11
Professional nurse (IB)	15	9
Professional midwife (SFE/ME)	9	5
Auxiliary midwife (AA)	34	19
General health worker (AIS)	29	16
Lab technician	15	9
Hospital chief accountant	1	1
Rather not say	8	5
**Years employed in Nouna district**		
<1	4	2
1-5	120	68
6-10	38	22
> 10	14	8

CSPS: Centre de Santé et Promotion Sociale.

CMA : Centre Médical avec Antenne Chirurgical.

AS : Attaché de Santé.

ICP : Infirmier Chef de Poste.

IDE : Infirmier Diplômé d’Etat.

IB : Infirmier Breveté.

SFE : Sage-Femme d’Etat.

ME : Magneticien d’Etat.

AA : Accoucheuse Auxiliaire.

AIS : Agent Itinérant de Santé.

In the fixed task, in which we presented the same choice set to all respondents, 13 (7%) chose among the two alternatives the one that was intended to be inferior (which offered a lower premium level, a capitation schedule of one payment per year, no donation of medical supplies and equipment, and no reimbursement of services fees). Models estimated with and without these respondents did not differ substantively and so, consistent with current practice, these respondents were retained in the main analysis [39].

The preferred payment options of health workers for the CBI provider payment attributes from Section 3 in the questionnaire are presented in Table 4. The majority of health workers preferred a baseline capitation of 1500 FCFA for children (59%), capitation payments made quarterly (42%), an annual CBI provision of medical materials and equipment (77%), 100% reimbursement of service fees (61%), the number of individuals enrolled as an indicator to determine the size of the RBF payment (54%), and a global payment to the facility health worker team as the payment mechanism for the RBF mechanism (59%).

**Table 4:** Health worker unrestricted payment preferences

**Payment attribute**	**Level**	**Percent (%)**
Capitation level	A	41
	B	59
Capitation Schedule	A	34
	B	24
	C	42
Provision of materials	A	11
	B	12
	C	77
Reimbursement of service fees	A	5
	B	34
	C	61
Results-based financing – indicator to determine size of payment	A	54
	B	30
	C	16
Results-based financing – Recipient	A	23
	B	59
	C	18

The odds ratios for selection of payment attributes estimated from the conditional logit models are shown in Table 5. In the main-effects model (Model 1), all attribute level alternatives were significantly different from zero except for household coverage as an evaluation indicator for the RBF mechanism (relative to individual coverage as an evaluation indicator). The main effects model had considerable overlap with the respondents' payment preferences (Table 4), both in preferences for attribute levels and signs of the odds ratios.

**Table 5:** Conditional logit model estimates

**Payment attribute alternatives**	**aOR**	**s.e.**	**% change**	**aOR**	**s.e.**	% change^*^	**aOR**	**s.e.**	**% change**
	**Model 1**			**Model 2**			**Model 3**		
Capitation plus subsidy	1.16***	(0.039)	16.0	1.05	(0.052)	5.0	1.15***	(0.044)	15.5
Capitation disbursed twice per year	1.18***	(0.046)	17.6	1.19**	(0.069)	18.7	1.12**	(0.046)	11.9
Capitation disbursed quarterly	1.08*	(0.041)	7.9	1.05	(0.061)	4.8	1.09*	(0.045)	9.5
Allocation of medical supplies	1.12**	(0.041)	11.7	1.11	(0.068)	11.2	1.09*	(0.045)	8.7
Allocation of medical supplies and equipment	1.47***	(0.054)	46.7	1.40***	(0.076)	40.2	1.44***	(0.058)	44.3
50% reimbursement of service fees	1.19***	(0.051)	19.0	1.27***	(0.081)	27.4	1.21***	(0.055)	21.4
100% reimbursement of service fees	1.49***	(0.075)	49.3	1.34***	(0.103)	34.5	1.41***	(0.077)	41.5
RBF^c^ indicator: households enrolled	0.97	(0.033)	−3.4	0.93	(0.049)	−7.3	0.97	(0.036)	−3.3
RBF indicator: greatest change in coverage	0.86***	(0.029)	−14.1	0.89*	(0.043)	−11.4	0.90**	(0.032)	−9.7
RBF recipient: global payment to health team	1.09*	(0.043)	9.0	1.19***	(0.063)	19.1	1.12**	(0.048)	12.4
RBF recipient: health facility management team	0.86***	(0.039)	−13.8	0.84**	(0.056)	−16.5	0.85**	(0.042)	−14.8
**Interaction terms**				**Male**			**Head nurse**		
Capitation plus subsidy	-	-	-	1.18*	(0.079)	18.4	1.02	(0.084)	2.2
Capitation disbursed twice per year	-	-	-	0.98	(0.076)	−1.9	1.37**	(0.151)	37.5
Capitation disbursed quarterly	-	-	-	1.06	(0.081)	5.5	0.92	(0.095)	−7.6
Allocation of medical supplies	-	-	-	1.01	(0.077)	1.2	1.18*	(0.101)	18.4
Allocation of medical supplies and equipment	-	-	-	1.09	(0.080)	8.6	1.10	(0.111)	10.2
50% reimbursement of service fees	-	-	-	0.89	(0.076)	−11.2	0.89	(0.120)	−10.7
100% reimbursement of service fees	-	-	-	1.20	(0.121)	20.1	1.41*	(0.188)	40.9
RBF indicator: households enrolled	-	-	-	1.07	(0.073)	7.0	1.02	(0.086)	1.7
RBF indicator: greatest change in coverage	-	-	-	0.95	(0.064)	−4.9	0.72***	(0.070)	−27.7
RBF recipient: global payment to health team	-	-	-	0.86	(0.067)	−13.8	0.84	(0.091)	−16.5
RBF recipient: health facility management team	-	-	-	1.05	(0.095)	5.3	1.10	(0.130)	9.6
**Number of respondents**	**176**			**176**			**176**		
**Number of observations**	**7392**			**7392**			**7392**		
**Log-likelihood**	**−4373**			**−4360**			**−4350**		
**Wald** ***X***^**2**^	**304.5**			**321.9**			**426.7**		
**Pseudo R-squared**	**0.080**			**0.083**			**0.085**		

aOR: adjusted odds ratio.

s.e.: standard error.

RBF: results-based financing.

^*^percent change in odds for unit increase in X.

In the main-effects model, 100% reimbursement of service fees (adjusted odds ratio (aOR) 1.49, p < 0.001) and donation of medical supplies and equipment (aOR 1.47, p < 0.001) had the largest effect on the probability of a payment alternative being chosen. Capitation payments being made twice per year had a larger effect on alternative selection than payments made four times per year (aOR 1.18, p < 0.001 vs. aOR 1.08, p = 0.046). For both the attributes “CBI donations of medical supplies and equipment” (level 2 aOR 1.12, p = 0.003; level 3 aOR 1.47, p < 0.001) and “reimbursement of medical fees” (level 2 aOR 1.19, p < 0.001; level 3 aOR 1.49, p < 0.001), an increase in attribute levels increased the odds of choosing a given alternative. For the RBF mechanism, both household coverage (aOR 0.97, p = 0.311) and an annual prize to the three facilities with the greatest increase in coverage (aOR 0.86, p < 0.001) decreased the odds of a given alternative being chosen (relative to the number of individuals enrolled as RBF indicator to determine payment size). For the RBF recipient, the odds of a payment mechanism alternative being selected decreased significantly when the local health facility management team was the recipient (aOR 0.86, p = 0.001), while a global payment to the entire facility health worker team increased the odds of selection of a payment alternative (aOR 1.09, p = 0.03) (relative to the CBI scheme pre-determining fund allocation among health facility staff). The only attribute level without a statistically significant effect on the selection of a payment alternative was household coverage as an indicator of evaluation for the RBF mechanism.

The estimates using random- and fixed-effects logit and random-effects probit models were not substantially different from the results of the conditional logit model, and provided poorer fits than\the conditional logit model (Table 6).

**Table 6:** Random- and fixed-effects models

	**Random-effects logit**		**Fixed-effects logit**		**Random-effects probit**	
	**Model 6**		**Model 7**		**Model 8**	
	**aOR**^**a**^	**s.e.**	**aOR**^**a**^	**s.e.**	**aOR**^**a**^	**s.e.**
Capitation plus subsidy	1.16***	(0.029)	1.16***	(0.029)	1.09***	(0.017)
Capitation disbursed semesterly	1.18***	(0.044)	1.18***	(0.044)	1.10***	(0.025)
Capitation disbursed quarterly	1.08*	(0.038)	1.08*	(0.038)	1.05*	(0.023)
Allocation of medical supplies	1.11**	(0.040)	1.12**	(0.040)	1.07**	(0.024)
Allocation of medical supplies and equipment	1.48***	(0.050)	1.47***	(0.050)	1.27***	(0.027)
50% reimbursement of service fees	1.20***	(0.042)	1.19***	(0.042)	1.12***	(0.024)
100% reimbursement of service fees	1.49***	(0.052)	1.49***	(0.052)	1.28***	(0.027)
RBF indicator: households enrolled	0.96	(0.034)	0.97	(0.035)	0.97	(0.022)
RBF indicator: greatest change in enrollment rate	0.86***	(0.031)	0.86***	(0.030)	0.91***	(0.020)
RBF recipient: global payment to health team	1.10**	(0.037)	1.09*	(0.037)	1.06**	(0.022)
RBF recipient: health facility management team	0.85***	(0.032)	0.86***	(0.032)	0.91***	(0.021)
Questionnaire version	0.98*	(0.008)			0.99*	(0.005)
**Number of observations**	**7,392**		**7,392**		**7,392**	
**Number of respondents**	**176**		**176**		**176**	
**Liklihood ratio** ***X*** **2**	**681.9**		**761.7**		**727.6**	
**Log-likelihood**	**−4738**		**−4373**		**−4738**	

aOR: adjusted odds ratio.

s.e.: standard error.

RBF: results-based financing.

For the model that estimated interaction terms between sex and payment attributes (Model 2), the only interaction term that had a significant effect on the selection of a payment mechanism was that between being male and increased capitation payment through child subsidies (aOR 1.18, p = 0.003). Being male had a positive effect on choosing an increased premium level. The model that tested interaction terms between professional qualifications and preferences for payment attributes (Model 3) included several odds ratios that were statistically significant. Being a facility head nurse had a positive effect on choosing a payment schedule of twice per year (aOR 1.37, p = 0.004), donation of medical supplies by the CBI (aOR 1.18, p = 0.047), and the full reimbursement of service fees (aOR 1.40, p = 0.01), but had a strong negative effect on the odds of selecting a payment mechanism alternative that included an annual prize for the greatest change in CBI coverage as an RBF evaluation indicator (aOR 0.72, p = 0.01). In the final two models (Models 4 and 5), none of the interaction terms significantly affected the odds of an alternative being selected. Estimates for these models are thus not presented in our results.

## Discussion

Ensuring that health workers are motivated to work towards the achievement of health system goals is a key component to successful health-sector interventions [40-42]. Previous studies have shown that health workers’ dissatisfaction with payment mechanisms in CBI can lead to low coverage, because health workers can influence the uptake of insurance in the population from which they draw their patients [13,26,43,44]. Our results provide new information about how health workers in Burkina Faso value different provider payment mechanisms in the context of a CBI scheme, where coverage has been low since the inception of the scheme, and there is strong evidence that health worker dissatisfaction with the scheme has contributed to the low coverage.

DCEs are a comparatively inexpensive approach to obtain data for health program planning and policy making [45-47]. DCEs have been used to elicit patient [46-48] and health worker [49-52] stated preferences in a variety of settings. In our study, we use DCE for the first time to elicit health worker preferences for provider payment mechanisms. Discrete choice experiments, in conjunction with focus group discussions and in-depth interviews to determine payment attributes, have the potential to be of great use in the process of choosing provider payment mechanisms in developing countries, potentially leading to better alignment between health worker incentives and health systems goals [21]. In our study, we find that reimbursement of service fees and CBI contributions for medical supplies and equipment were the attributes of the insurance payment mechanism valued most by health workers. For a proposed RBF mechanism linking health worker financial incentives to insurance coverage, health workers significantly preferred to be paid a flat payment for each individual who enrolled in their catchment area as opposed to a competitive prize for the health centers that achieved the largest coverage gains. Health workers were also strongly opposed to payment of the RBF to the health-facility account managed by the local health management committee.

### Capitation payment

Capitation payment may help to control costs by transferring health-expenditure risk to the health workers or facilities [53]. In the case of the CBI scheme in Nouna, health expenditure deficits incurred by contracted facilities were reimbursed at the end of each year. As a result, health-expenditure risk was not transferred to facilities, but facilities commonly suffered negative consequences of temporary revenue shortfalls, as they were not able to restock drugs or supplies until the annual reimbursement was paid. In previous years, due to depleted funds several facilities had been obligated to restock drugs on credit from the District Health Office, and were only able to pay off their loans after reimbursement by the CBI [23,25]. It is likely that replacing the annual capitation payment with a bi-annual one – as strongly preferred by the health workers in our study – will reduce the probability of facility bankruptcy and drug stock-outs. Surprisingly, controlling for other payment attributes, respondents preferred payments in two rather than in four installments. One reason for this may be the fact that enrollment continues to be limited and premiums low; leading to overall small capitation payments. If capitation payments were divided into four installments, they may be considered too insignificant for planning purchases or investments, and setting up dedicated savings accounts for installments may carry substantial transaction costs.

### Service fees

In a previous qualitative study on health worker perceptions of the CBI scheme, providers expressed concern that increased CBI coverage would lead to higher healthcare utilization rates [23]. In the past, high healthcare utilization rates among enrollees led to challenges in ensuring uninterrupted basic medical supplies for consultations and services. As consultation and service fees are neither directly paid by CBI enrollees nor reimbursed by the insurance scheme, health workers feared that any increase in CBI coverage of the population from which their facilities drew their patients could decrease facility revenue, leading to less disposable income to purchase basic medical supplies [23]. Our DCE provides further evidence for this fear: reimbursement of service fees and an annual provision of medical supplies and materials by the CBI scheme had the greatest effect on whether a payment alternative was chosen or not, leading to the recommendation to diversify provider payment in the CBI to include these mechanisms.

### Health worker characteristics and preferences

Our DCE analysis shows that sex does not play a particular role in health worker preferences for CBI payment attributes, except in the preference for an increase in the capitation payment for children. Men (n = 103, 59%) significantly preferred an increase in the capitation per child, while women (n = 73, 41%) did not. This difference may be due to certain respondents misinterpreting the increase in capitation payments as an increase in premium payments, borne by individual enrollees, while in fact the increase in capitation payments would be a result of external subsidization for children, and not a direct increase in premium levels for enrollees. If this were the case, it might imply that women were more likely to fear that such an increase could in the long-term reduce children’s access to health insurance and healthcare.

The CBI payment preferences for head nurses (n = 32, 18%) were significantly different from other health workers for several payment attributes. Head nurses significantly preferred capitation payments made twice instead of once per year, while other health workers did not share this preference. Head nurses also preferred RBF payments to be paid directly to the health worker team in a lump-sum payment, as opposed to a method of distribution predetermined by the CBI management committee or being paid to the local management committee. Furthermore, head nurses were particularly opposed to a competition-based RBF evaluation mechanism. These differences in payment preferences between head nurses and other health workers may stem from head nurses’ particular awareness of factors affecting the financial status of their facilities. Facility head nurses are also likely to understand the functioning of the CBI payment mechanism better than other health workers.

It is plausible that this increased knowledge, in particular the abovementioned concerns regarding the current capitation payment schedule, may contribute to the expressed preference for payments being made in both April and July. Regarding the preference for RBF payments being made directly to the health worker team, head nurses may view the opposing alternatives as leading to a reduction in their autonomy in deciding how to utilize these new payments.

Neither the level of care of the facility where the respondent worked nor employment in the current CBI implementation zone (rather than outside the zone) significantly affected the type of payment mechanism preferred by health workers in our study. Previous studies have noted that health worker preferences and motivation are strongly affected by past experiences with insurance and the level of care at the facility where a health worker is employed [54]. It is likely that we did not find such effects, because our variables on level of care and CBI experience only capture the situation at the time of the survey and not past work experiences, which for many health workers in this community have included levels of care other than the levels of their current facilities and employment both with and without CBI contracts.

### Policy changes based on the DCE results

Upon completing the analysis of the DCE data in September 2010, the authors conducted a series of meetings with local decision-makers involved with the CBI scheme in Nouna, in order to disseminate results and discuss policy implications. Meetings were held with the CBI Management Team, the CBI Community Representation Committee, the Scientific Committee of Nouna Health Research Centre, the District Health Office, senior staff at the district hospital, and the head nurses from the 13 primary-care facilities contracted with the scheme. Presentations were made to each stakeholder highlighting the key results of the DCE. These presentations were followed by brainstorming and discussion sessions on how to revise the payment mechanism for the upcoming enrollment campaign in 2011. Based on the discussions of the DCE findings in these meetings, a new payment mechanism, which closely reflects the health-worker preferences for payment mechanisms elicited in the DCE, was adopted in October 2010 (see Table 7). As of January 2011, health workers are paid in two installments (April and July each year); capitation levels for children have increased to 1,500 FCFA ($3 USD), with the additional 1,000 FCFA added as a subsidy by an external donor; and consultation fees are fully reimbursed at the end of each year. No provision of medical supplies is provided by the scheme, as increased revenue generated from the reimbursement of consultation fees are now used to cover the cost of supplies. Payments for the RBF are calculated based on each individual who enrolls in the primary-care facility catchment area during the annual enrollment period. Facilities are now paid an additional 200 FCFA ($0.40 USD) per new enrollee and 100 FCFA ($0.20 USD) per re-enrollee. During the stakeholder discussions, several participants stressed the importance of including the local health management committee in the payment process, and that the health worker team should not be entitled to 100% of the RBF payments. Thus the decision was made to earmark 25% of the RBF payments for facility improvement funds, which would be managed by the local health management committee.

**Table 7:** Changes in CBI payment mechanisms based on the results of this study

**Payment attribute**	**Previous payment mechanism (2004–2010)**	**New payment mechanism (2011)**
Capitation payment level	The enrollment premium was 500 FCFA ($1 USD) for children under 15 and 1500 FCFA ($3 USD) for adults 15 and older.	The enrollment premium is 500 FCFA ($1 USD) for children under 15 and 1500 FCFA ($3 USD) for adults 15 and older. A 1000 FCFA subsidy for children is added for each child enrolled, resulting a capitation level of 1500 FCFA for children and 1500 FCFA for adults.
Capitation payment schedule	The capitation was paid once a year, normally in July or August.	The capitation is paid twice per year, once in April (after the first three months of the enrollment period) and once in July (after the closing of the enrollment period).
Allocation of medical supplies and equipment	No medical supplies or medical equipment were provided to the facilities that are contracted with the CBI scheme.	No change from previous mechanism.
Reimbursement of consultation and service fees	None.	100% of consultation fees of CBI enrollees are calculated at the end of the calendar year and reimbursed to health facilities during the first quarter of the following calendar year.
Capitation deficit reimbursement	If enrollees were prescribed more drugs than the capitation covers, the deficit was calculated at the end of each calendar year and reimbursed during the first quarter of the following calendar year.	No change from previous mechanism.
Results-based financing (RBF) provider payment mechanism	None.	For each individual enrolled in a primary-care facility (CSPS) catchment area, the primary-care facilities are paid 200 FCFA ($0.40 USD) per new enrollee and 100 FCFA ($0.20 USD) per re-enrollee. Payments are divided between a direct global payment to the facility health worker team (75%) and the health facility account (25%), and will be paid in April and July. The secondary-care facilities (CMA) do not receive any RBF payments.

FCFA: Franc Communauté Financière Africaine, the local currency used in Burkina Faso. 500 FCFA = $1 USD.

CBI: Community-based health insurance.

CSPS: Centre de Santé et Promotion Sociale.

CMA: Centre Médical avec Antenne Chirurgical.

While the new payment mechanisms were introduced in January 2011, the CBI Management Unit decided to introduce it in a staggered fashion, first in half (7) of the primary-care facilities and only later in all of the 13 primary-care facilities and the district hospital. The initial assignment to the change in provider payment mechanisms was randomized at the facility level. Prior to introducing the new payment mechanisms, alongside the discrete choice experiment, in-depth interviews and a quantitative “satisfaction survey” were conducted to assess workers’ satisfaction in relation to the applied payment mechanisms. Facility-based patient exit interviews were also conducted to measure client satisfaction and health worker behavior during consultations, with particular focus on whether health workers promoted CBI during patient visits. The randomized assignment will provide the opportunity to confirm the expectations raised by this study and to measure the effect of the changes on CBI coverage, provider satisfaction, and patient satisfaction in a randomized controlled experiment.

### Study limitations

We note a few potential limitations of our study. Firstly, some respondents may have misunderstood the payment attribute level related to premium levels for children, because a large proportion of respondents chose an attribute level that was intended to be inferior. Given that the proposed increase in capitation payments for children was to be subsidized by an external donor, the higher payment level should have received 100% support. Another explanation for the large proportion of respondents who chose the attribute level intended to be inferior is that respondents were fearful that relying on such subsidization could generate an unsustainable dependency on external support. If the subsidy were to end after a few years, enrollees themselves would potentially become responsible for the higher payments.

Furthermore, as in any study conducted in a particular community, our results may not be applicable outside this particular setting, which has had a distinct history with experiences with alternative health insurance structures that other communities in Burkina Faso are lacking. Future studies need to examine whether health worker preferences for provider payment mechanisms are similar in other communities.

## Conclusions

Health worker support is a key component to CBI sustainability and success. It may be possible to increase this support by revising the CBI payment mechanism to align health worker incentives with CBI objectives, such as providing quality care and expanding coverage, and by financially motivating health workers to increase efforts to promote and support the scheme. Our study demonstrates that it is feasible to elicit health worker preferences for provider payment mechanisms in a rural district in a sub-Saharan African country. We find that reimbursement of service fees and CBI contributions for medical supplies and equipment were the attributes of the insurance payment system valued most by health workers. Based on our results, the provider payment mechanisms in the CBI scheme in Nouna health district, Burkina Faso, were recently changed. These changes are expected to increase health worker support for the CBI scheme and to lead to increased CBI coverage, improving the long-term performance of the scheme. Ongoing studies will establish whether these expectations will be met, and will thus provide an opportunity to validate our findings in their use for health policy.

## Abbreviations

CBI, community-based health insurance; AMBC, assurance maladie à base communautiare; DCE, discrete choice experiment; RBF, results-based financing; FCFA, Franc Communauté Financière Africaine CFA; USD, United States Dollars; AOR, adjusted odds ratio; SE, standard error.

## Competing interests

The authors declare that they have no competing interests.

## Authors’ contributions

PR was the principal investigator and wrote the paper. TB made substantial contributions to the analysis and interpretation of the data. He also reviewed the first draft. AS, GS, BB, AE, and RS contributed significantly to management of the study throughout the data collection process. RS made substantial contributions to the study design and development of the research question. All authors read and approved the final manuscript.

## Pre-publication history

The pre-publication history for this paper can be accessed here:

http://www.biomedcentral.com/1472-6963/12/159/prepub
